# ProX from marine *Synechococcus* spp. show a sole preference for glycine‐betaine with differential affinity between ecotypes

**DOI:** 10.1111/1462-2920.16168

**Published:** 2022-08-28

**Authors:** Benjamin A. Ford, Pramita Ranjit, Bridget C. Mabbutt, Ian T. Paulsen, Bhumika S. Shah

**Affiliations:** ^1^ School of Natural Sciences Macquarie University Sydney Australia; ^2^ ARC Centre of Excellence in Synthetic Biology Macquarie University Sydney Australia

## Abstract

Osmotic stress, caused by high or fluctuating salt concentrations, is a crucial abiotic factor affecting microbial growth in aquatic habitats. Many organisms utilize common responses to osmotic stress, generally requiring active extrusion of toxic inorganic ions and accumulation of compatible solutes to protect cellular machinery. We heterologously expressed and purified predicted osmoprotectant, proline/glycine betaine‐binding proteins (ProX) from two phylogenetically distinct *Synechococcus* spp. MITS9220 and WH8102. Homologues of this protein are conserved only among *Prochlorococcus* LLIV and *Synechococcus* clade I, III and CRD1 strains. Our biophysical characterization show *Synechococcus* ProX exists as a dimer, with specificity solely for glycine betaine but not to other osmoprotectants tested. We discovered that MITS9220_ProX has a 10‐fold higher affinity to glycine betaine than WH8102_ProX, which is further elevated (24‐fold) in high salt conditions. The stronger affinity and effect of ionic strength on MITS9220_ProX glycine betaine binding but not on WH8102_ProX alludes to a novel regulatory mechanism, providing critical functional insights into the phylogenetic divergence of picocyanobacterial ProX proteins that may be necessary for their ecological success.

## INTRODUCTION

Abiotic factors such as high and fluctuating salt concentrations constrain microbial growth in aquatic habitats (Scanlan et al., 2009). Organisms faced with osmotic stress have evolved common response mechanisms: active extrusion of inorganic ions and the accumulation of compatible solutes (Hagemann, 2011; Scanlan et al., 2009). These compatible solutes are usually small molecular weight compounds, such as sucrose, trehalose, glucosylglycerol and betaines, accumulated in high concentrations to maintain cellular turgor without compromising cellular metabolism. These compounds have also been shown to alter the water structure around macromolecules, providing direct protection to cellular machinery against denaturation caused by desiccation or high‐salt concentrations (McNeil et al., 1999).

Seasonal variability, upwelling events, vertical mixing, riverine discharge, and haloclines are commonly known factors influencing ocean salinity (Aagaard et al., 1981; Brugler et al., 2014; Hátún et al., 2005). However, anthropogenic effects such as the input of hypersaline release from desalination plants (Belkin et al., 2015) and the impact of climate change on the intensification of water cycles (Durack et al., 2012) are also likely to continue to change local salinity profiles.

Cyanobacteria are an ancient bacterial lineage that has colonized many diverse habitats, including freshwater, estuarine, and open ocean environments (Blankenship, 2017; Lauro et al., 2009; Scanlan et al., 2009). Most picocyanobacteria cannot withstand dramatic changes in salt concentrations as they have adapted to relatively stable salt concentrations over long periods (Scanlan et al., 2009). This assumption is underpinned by the absence of an aquaporin gene, *aqpZ* (Cluster CK_6866), from almost all picocyanobacteria (Scanlan et al., 2009).

Among cyanobacteria, the influence of environment, salt tolerance, and preference for compatible solutes are highly correlated (Hagemann, 2011; Reed et al., 1986). Freshwater strains (halotolerant to 0.6 M NaCl) accumulate disaccharides such as sucrose and trehalose (Reed et al., 1986), whereas moderately halotolerant (up to 1.7 M NaCl) strains accumulate glucosylglycerol and ‐glycerate (Reed et al., 1986). Halophilic strains, capable of tolerating up to 3 M NaCl concentrations, usually accumulate zwitterionic betaines, such as trimethyl glycine, ‐glutamate, ‐proline, or ‐serine (Pade et al., 2016; Reed et al., 1986; Scanlan et al., 2009).

The ability to synthesize specific compatible solutes, such as sucrose via the sucrose‐phosphate synthase pathway, is thought to be a typical metabolic response in cyanobacteria (Baran et al., 2013; Hagemann, 2011; Lu et al., 2006), with several freshwater strains known to have horizontally acquired the requisite genes (Tanabe et al., 2018). It is recognized that no marine picocyanobacterial strains can synthesize trehalose (Scanlan et al., 2009). While glycine betaine synthesis is unexpected in most marine picocyanobacteria strains due to moderate salinity levels, proteins similar to methylating enzymes (Gbmt1/Gbmt2) responsible for glycine betaine synthesis in halotolerant (Waditee et al., 2003) and freshwater cyanobacterium (Waditee et al., 2005) are identified in some marine picocyanobacterial genomes (Scanlan et al., 2009). Uptake of compatible solutes by primary active ABC transporters and symporters is considered a preferred metabolic strategy employed by heterotrophic marine bacteria (Hagemann, 2011) and halophilic cyanobacteria (Aikawa et al., 2019). The de novo synthesis of some classes of osmoprotectants, such as betaines, also requires investment of nutrient (nitrogen) resources which can be growth limiting (Scanlan et al., 2009).

All sequenced marine *Synechococcus* (*n* = 48) strains possess an ABC transporter predicted to uptake glucosylglycerol/trehalose/sucrose (*Ggt*, Cluster CK_1455). However, only a select number (*n* = 28) contains predicted high‐affinity uptake machinery for the compatible solute proline/glycine betaine (*proU* operon, Cluster CK_1944). Experimental evidence has shown that synthesized compatible solutes such as glutamate and glucosylglycerol are rapidly turned over in cyanobacteria (Baran et al., 2017); thus, the presence of the *proU* operon might be due to the re‐uptake of leaked compatible solutes into the periplasmic space or hypersalinity resulting from intense diurnal surface evaporation (Scanlan et al., 2009). Biochemical validation of related *proU* operon systems (e.g. in *Escherichia coli*) shows that the specificity imparted by the substrate‐binding protein (SBP) can tune the transporter to recognize multiple compatible solutes, including both glycine‐ and proline betaine (Schiefner, Breed, et al., 2004). In some picocyanobacterial strains (e.g. *Prochlorococcus* sp. MIT9313), possible alternative substrates of the *proU* system include mimics of quaternary amines such as dimethylsulfopropionate (DMSP), which may instead provide a source of sulfur (Hagemann, 2011; Scanlan et al., 2009), rather than function to uptake osmoprotectants. Similarly, in other bacteria (e.g. *Bacillus subtilis*), there is evidence that the conserved molecular scaffold of the SBP has been co‐opted to recognize non‐canonical substrates such as organoarsenate compounds (Hoffmann et al., 2018).

Given the conserved molecular scaffold of the SBP componentry and its potential to recognize a diverse range of substrates, the question of the biological role of the *proU* operon requires further investigation. We undertook biophysical characterization of the SBP (annotated ProX; Cluster CK_1944) encoded in the *proU* operon from two distinct marine *Synechococcus* strains (*Synechococcus* spp. MITS9220 and WH8102) to provide a detailed understanding of its ligand preferences and salt‐dependence of ligand interactions.

## EXPERIMENTAL PROCEDURES

### Genomic and phylogenetic analyses

The Cyanorak database (www.sb-roscoff.fr/cyanorak) is a repository for 97 sequenced cyanobacterial genomes (48 unique *Synechococcus*, 43 *Prochlorococcus* and 6 *Cyanobium*) (Doré et al., 2020; Garczarek et al., 2021). Genes are organized into clusters based on their sequence similarity. One gene cluster, CK_00001944, comprises 33 orthologous sequences, of which 28 arise from *Synechococcus* strains, annotated to bind glycine betaine/proline. Their annotation is based on mild sequence homology (~30%) to a verified glycine betaine‐binding protein isolated from *Lactococcus lactis* (Wolters et al., 2010). Phylogenetic analysis was performed using a modified method given by Wilding et al. (2017). Briefly, the 33 orthologous sequences (average sequence ID = 70%) from CK_1944 were used to compute a preliminary multiple sequence alignment, calculated using the L‐iNS‐I option of MAFFT (Katoh & Standley, 2013). The phylogenetic tree was inferred using IQ‐Tree (Nguyen et al., 2015), with the optimal model, using the TESTONLY option, found to be JTT + G4. The inferred maximum likelihood model was used to generate a final phylogenetic tree and visualized using iTOL (Letunic & Bork, 2019).

The geographic distribution and environmental context of *proX* genes of interest were analysed using the Ocean Gene Atlas tool (Villar et al., 2018) to visualize data from the *Tara* Oceans expeditions (Sunagawa et al., 2020). Briefly, the nucleotide sequence for each gene was used to search the Ocean Gene Atlas against the ‘OM_RGC_v2_metaG’ catalogue using the default parameters and a filter expectation value (e‐value) e^−10^. The results were visualized by taxonomy to ensure only *Synechococcus* genes of interest were captured. Plots were then viewed using salinity as the environmental parameter of interest.

### Protein expression and purification

The genes *MITS9220_00385* and *WH8102_01917* are annotated to encode a putative glycine betaine‐binding protein, referred to as MITS9220_ProX and WH8102_ProX, respectively. MITS9220_ProX is derived from *Synechococcus sp*. MITS9220 collected from equatorial Pacific waters (0°0′0″ N, 140°0′0″), while WH8102_ProX instead originates from *Synechococcus* sp. WH8102 collected from the Caribbean Sea (22°28′59″, 65°35′59″). ProX sequences (obtained from the Cyanorak database) (Garczarek et al., 2021) were analysed using the SignalP4.0 server (Petersen et al., 2011), which predicted the presence of a twin‐arginine (Tat) signal peptide. This was removed to facilitate purification. The required genes (~900 bp) were then PCR‐amplified from genomic DNA (extracted using the CTAB/phenol‐chloroform method) from laboratory isolates maintained at Macquarie University (Paulsen Laboratory), incorporating vector‐specific (pET15‐b, Novagen) overhang regions. Ligation‐independent cloning (Clontech) (Aslanidis & de Jong, 1990) into the pET15‐b vector (Novagen) was carried out using *BamHI* and *NdeI* restriction sites to incorporate an N‐terminal hexahistidine tag with a thrombin cleavage site. Transformants were screened by colony PCR, and the sequence of the cloned product was verified (Macrogen).

Following plasmid transformation, *E. coli* DE3 BL21 cells were grown (500 ml cultures in 2 L baffled flasks) at 25°C, 200 rpm for all preparations. Growth occurred for 24 h using the autoinduction method (Studier, 2005). Cells were harvested by centrifugation (4000 *g*), resuspended in Buffer A: HEPES (50 mM, pH 7.4), NaCl (500 mM), and glycerol (5% v/v) with the addition of imidazole (5 mM). Cells were lysed with a single freeze–thaw step (liquid N_2_) and sonication on ice. Clarified lysate (30 ml) was loaded with a peristaltic pump onto a prepacked Ni‐NTA column (1 ml, GE Healthcare) pre‐equilibrated in the same buffer. The protein product was eluted by direct addition of five column volumes of imidazole (500 mM) in Buffer A. Eluate fractions were pooled and desalted using size‐exclusion chromatography (SEC) (Superdex HiLoad 200 16/600 column, GE Healthcare) in Buffer A. Reducing agent tris(2‐carboxyethyl)phosphine (TCEP, 0.5 mM) and glycerol (5% v/v) was included in all buffers for protein purification.

Protein‐containing fractions were pooled, concentrated using centrifugation (Centricon 10 kDa MWCO), and snap‐frozen (50 μl aliquots) in liquid N_2_. The purity of the recovered His_6_‐tagged product was verified using SDS‐PAGE, showing a distinct band at ~30 kDa when visualized with Coomassie blue dye.

### Analytical size‐exclusion chromatography

The evaluation of native ProX protein mass in solution was carried out using analytical SEC procedures on a Superdex 200 10/300 GL column (GE‐Healthcare) equilibrated in Buffer A. Calibration of elution times was carried out using commercial size standards (GE‐Healthcare), and the void volume (*V*_0_) estimated using blue dextran. Partition coefficients (*K*_av_) were calculated from elution volumes and used to derive a plot of *K*_av_ against log(*M*_R_) to allow the interpolation of unknown masses based on elution volume. The line of best fit was given as log(*M*_*R*_) *= −*3.748(*K*_av_) + 6.593, with a correlation coefficient (*R* ^2^) of 0.9957.

### Differential scanning fluorimetry

Commercially available libraries of small molecules were tested as potential binding partners to ProX by recording differential scanning fluorimetry (DSF) responses over a thermal gradient (Neisen et al., 2007). SYPRO Orange dye (Invitrogen) was used to monitor fold integrity in discrete protein mixtures by measuring fluorescence at 590 nm following excitation at 485 nm using a Stratagene MX3000P q‐PCR instrument. Compounds tested in 96‐well blocks incorporated common protein stabilizing ligands (HR2‐096 Silver Bullets, Hampton Research) and single‐molecule osmolyte sources (MicroPlate PM9, Biolog Inc). The concentration of these compounds following reaction set‐up was ~4 μM, and, therefore below the concentration required to effect complete saturation of the binding pocket. Each condition was tested in triplicate, with each plate containing a control well with no additive.

Thermal melt curves were analysed using the analysis template provided (Neisen et al., 2007) and fitting of Boltzmann distribution (GraphPad Prism) for the determination of mid‐point thermal melt temperatures (Bai et al., 2019). The only condition (Silver Bullets well #D8) leading to a significant increase (>3°C) in melting temperature was deconvoluted to identify the single component leading to such a change and repeated in triplicate.

### Determination of ProX substrate binding affinity

The DSF assay was modified to elucidate binding kinetics, following a published protocol specifically outlining this application, and validated using SBPs (Bai et al., 2019). Briefly, a range of protein concentrations were tested (2–20 μM) to determine the optimal signal‐to‐noise ratio while requiring a low protein concentration (5 μM). Protein solutions were prepared in Buffer A at a concentration of 10 μM, with a 50× concentration of SYPRO Orange dye. Working stocks of ligands were prepared independently (in Buffer A) at the following concentrations: 0.4, 0.8, 1.6, 3.2, 6.4, 12.8, 25, 50, 100, 200, 2000 μM. ProX protein and ligands (10 μl each) were both delivered in triplicate into a 96‐well plate (final volume 20 μl). Plates were centrifuged in a benchtop plate spinner (1 min) to ensure sample homogeneity before recording fluorescence data across the thermal ramp as previously described.

Data were processed using two independent software packages: the provided Python package (Bai et al., 2019) and the web‐based FoldAffinity tool (Niebling et al., 2021) to ensure their consistency and determine affinity values. The final values reported were taken from FoldAffinity. Processed results were then plotted (GraphPad Prism) to generate publication‐quality figures of the processed data.

### Protein structure modelling

Protein structures were predicted using AlphaFold (Jumper et al., 2021) through the online ColabFold interface (Mirdita et al., 2022). Briefly, the query sequences (full length) were uploaded to the interface, and the structure prediction was performed using the default parameters: multiple sequence alignment using the MMSeqs2 (UniRef + Environmental) database; model type ‘auto’; pair mode ‘unpaired + paired’; and a number of recycles = 3. The top five models were manually inspected to identify regions where the structural predictions disagreed. The top‐ranked models for each target were then analysed further by comparing these to known, validated exemplars of functional relatives (Table 2) to identify critical conserved structural features involved in ligand binding.

## RESULTS AND DISCUSSION

### Distinct delineation of picocyanobacteria ProX proteins

**FIGURE 1:**
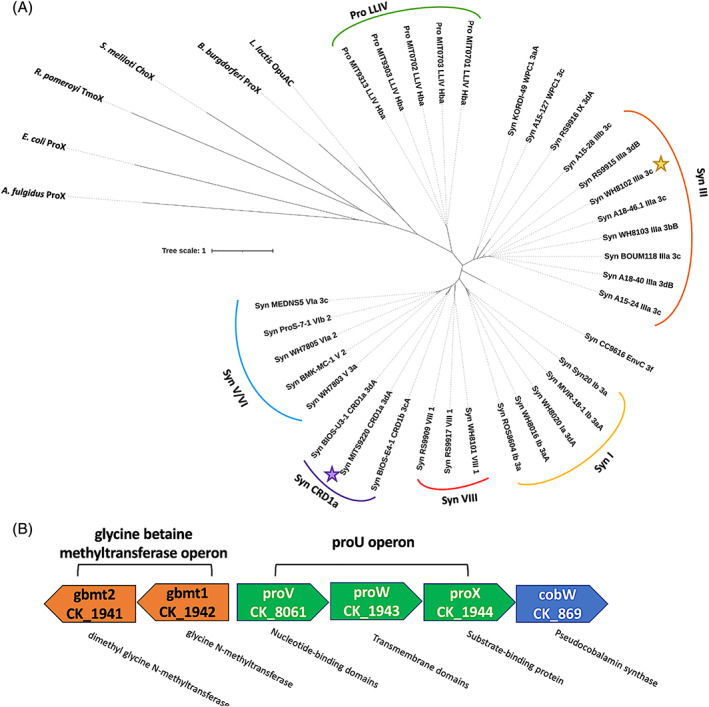
Phylogenetic analyses of picocyanobacterial ProX (CK_1944) sequences. (A) The amino acid sequences of picocyanobacterial ProX and diverse osmoprotectant‐binding proteins were aligned using MAFFT (Katoh & Standley, 2013), and the phylogenetic tree was inferred using IQ‐Tree (Nguyen et al., 2015). The final phylogenetic tree was visualized using iTOL (Letunic & Bork, 2019). The two *Synechococcus* ProX proteins, MITS9220_ProX and WH8102_ProX, functionally characterized in this study, are highlighted on the tree. Syn and Pro denote *Synechococcus* and *Prochlorococcus*, respectively. (B) The typical genomic context for the SBP (denoted ProX) with its related transporter elements (green) and compatible solute biosynthesis machinery (orange). Genes are numbered according to their Cyanorak cluster designation (Garczarek et al., 2021).

The Cyanorak cluster CK_1944 (Garczarek et al., 2021) comprises 33 orthologous ProX sequences, of which 28 arise from *Synechococcus* strains and 5 from *Prochlorococcus* strains. Members of this cluster are predicted to be a glycine/proline betaine‐binding protein (GBP), based on sequence similarity to other GBPs, such as the lactococcal OpuAC (Wolters et al., 2010) and conservation of their broader genomic context. A phylogenetic tree incorporating the Cyanorak ProX sequences and diverse osmoprotectant binding proteins is depicted in Figure 1A. The tree shows a clear taxonomic delineation of ProX protein between *Prochlorococcus* and *Synechococcus* strains. Notably, only one low‐light *Prochlorococcus* clade (LLIV) possesses the ProX protein (*n* = 5). The members of this clade are found in the lower euphotic zone in tropical and subtropical regions (Huang et al., 2012; Scanlan et al., 2009). Notably, the absence of ProX from other marine picocyanobacterial strains in the Cyanorak database directly correlates with the absence of the glycine betaine synthesis proteins, Gbmt1 (cluster CK_1941) and Gbmt2 (cluster CK_1942).

The *Synechococcus* ProX sequences group broadly into two clusters: one consisting almost exclusively of *Synechococcus* Clade III isolates (including *Synechococcus* sp. WH8102), mainly found in tropical and subtropical oligotrophic waters, and the second with representatives from Clades I, V, VI, VIII, and CRD1 (including *Synechococcus* sp. MITS9220). Isolates from Clade CRD1 are found in the sub‐equatorial Pacific, particularly in upwelling regions (Saito et al., 2005). Members of Clade I are found in temperate, coastal, and mesotrophic waters (Zwirglmaier et al., 2008). Members of most other clades are more widely distributed, with Clades V/VI corresponding with upwelling zones and those in Clade VIII with euryhaline regions (Dufresne et al., 2008; Zwirglmaier et al., 2008).

Analysis of the genomic context (Figure 1B) indicates ProX proteins conform with the typical organization of SBPs, encoded within an operon containing their associated transport machinery (Thomas, 2010). Further, *proX* is found adjacent to the *gbmt1/gbmt2* operon (up‐ or downstream), responsible for glycine betaine synthesis (methylation of glycine), forming the basis for their annotation as glycine/proline betaine‐binding proteins (Mellies et al., 1994; Wolters et al., 2010). An adjacent pseudo‐cobalamin synthesis gene, *cobW*, involved in synthesizing an alternative form of cobalamin utilized by cyanobacteria (Heal et al., 2017), is likely not functionally linked to genes in these other operons.

**FIGURE 2:**
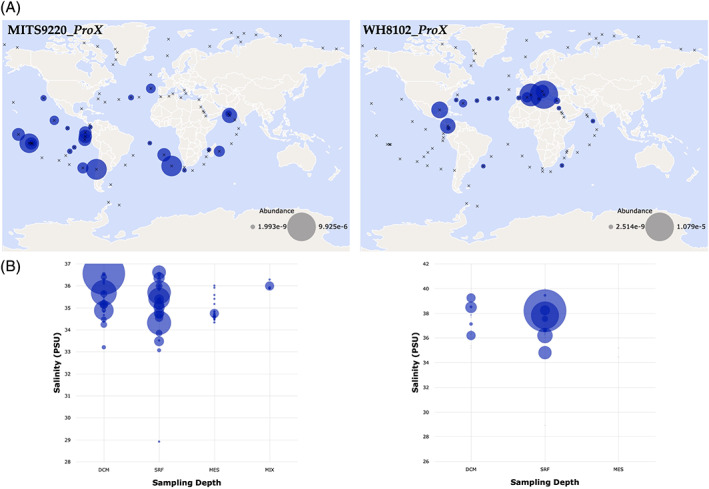
Ecological distribution of MITS9220 *proX* and WH8102 *proX* homologues. (A) The environmental abundance for MITS9220_*ProX* (left) and WH8102_*ProX* (right) homologues extracted from the Tara Oceans MetaG dataset (Sunagawa et al., 2020). The abundance of the genes of interest is plotted for surface waters as a function of measured salinity, with circle size corresponding to the measured abundance at a particular sampling site. Sampling sites are denoted by an ‘X'. (B) The corresponding bubble plot for each gene across all four sampling depths as a function of measured salinity for MITS9220_*ProX* (left) and WH8102_*ProX* (right) homologues. DCM, deep chlorophyll maximum; MES, mesopelagic zone; MIX, marine epipelagic mixed layers; SRF, surface waters. MIX data are not available for WH8102_ProX. Salinity is measured in PSU (practical salinity units).

The *Synechococcus* MITS9220 and WH8102 *proX* gene sequences were used to search the Ocean Gene Atlas (Villar et al., 2018), an interactive display of the data collected across *Tara* Oceans sampling voyages (Sunagawa et al., 2020). This identifies the homologues of the genes of interest and allows their geographical distribution to be plotted according to various ecological parameters. These data immediately highlighted geographical differences between the abundance of each gene within the *Tara* Oceans marine metagenomes (Figure 2A): MITS9220 *proX* and close homologues (>75% nucleotide sequence identity) displayed a more widespread distribution, particularly across various sampling sites around the Pacific Ocean, encompassing a range of coastal and open‐ocean conditions. In contrast, WH8102 *proX* and its homologues (>75% nucleotide sequence identity) were isolated predominately to surface waters in areas such as the Mediterranean, possibly reflective of the localized salinity profiles in this area. Given that its functional annotation implies *proX* is involved in response to osmotic stress, geographical distributions were plotted against salinity (Figure 2). Visualizing this data on a bubble plot (Figure 2B) indicates that the salinity profiles encompassed by MITS9220 *proX* and its homologues are generally lower and across a broader range. In contrast, the salinity profiles of WH8102 *proX* and its homologues are comparably higher and less expansive, lending further support to phylogenetic delineation likely correlating to their eco‐physiological success. Similar geographical distribution differences between the abundance of MITS9220/WH8102 *proX* and their close homologues were observed in the *Tara* Oceans marine metatrancriptomes (see Figure S1).

### ProX proteins from *Synechococcus* spp. are dimeric in solution

The genes encoding ProX proteins from two *Synechococcus* strains (MITS9220 and WH8102) originating from distinct ocean environments, hereafter referred to as MITS9220_ProX and WH8102_ProX, were successfully produced as a monodisperse product in >95% purity via heterologous expression in *E. coli*. Typical protein titres varied between 5 and 20 mg L^−1^ using the large‐scale autoinduction method (Studier, 2005). Polishing steps, using preparative SEC, showed both ProX proteins favour a dimeric state, with an absence of aggregation indicating them to be stable within the solution (Figure S2). That both ProX proteins favour, a dimeric solution state is a clear divergence from their closest characterized sequence relative, lactococcal OpuAC (sequence ID = 28.7%) and other archetypal family members (ProX, *E. coli*; Schiefner, Holtmann, et al., 2004), which instead favour a monomeric assembly typical of that expected for an SBP (Wolters et al., 2010). A few SBPs have been experimentally shown to exist as dimers, such as the α‐keto acid‐binding proteins (Cuneo, Changela, et al., 2008; Li et al., 2017), where dimer–monomer interconversion is tied to ligand binding; and the *lac* repressor protein family, where protein dimerization and ligand binding exert control over DNA‐binding (Lewis et al., 1996). The *Synechococcus* ProX proteins join a limited repertoire of dimeric SBPs, many of which have evolved allosteric mechanisms that modulate their function, including in carbohydrate‐ (Culurgioni et al., 2017; Cuneo, Beese, et al., 2008), arginine‐ (Ruggiero et al., 2014), and phosphate‐binding proteins (Alicea et al., 2011).

### *Synechococcus* spp. ProX proteins show specificity for glycine betaine

**FIGURE 3:**
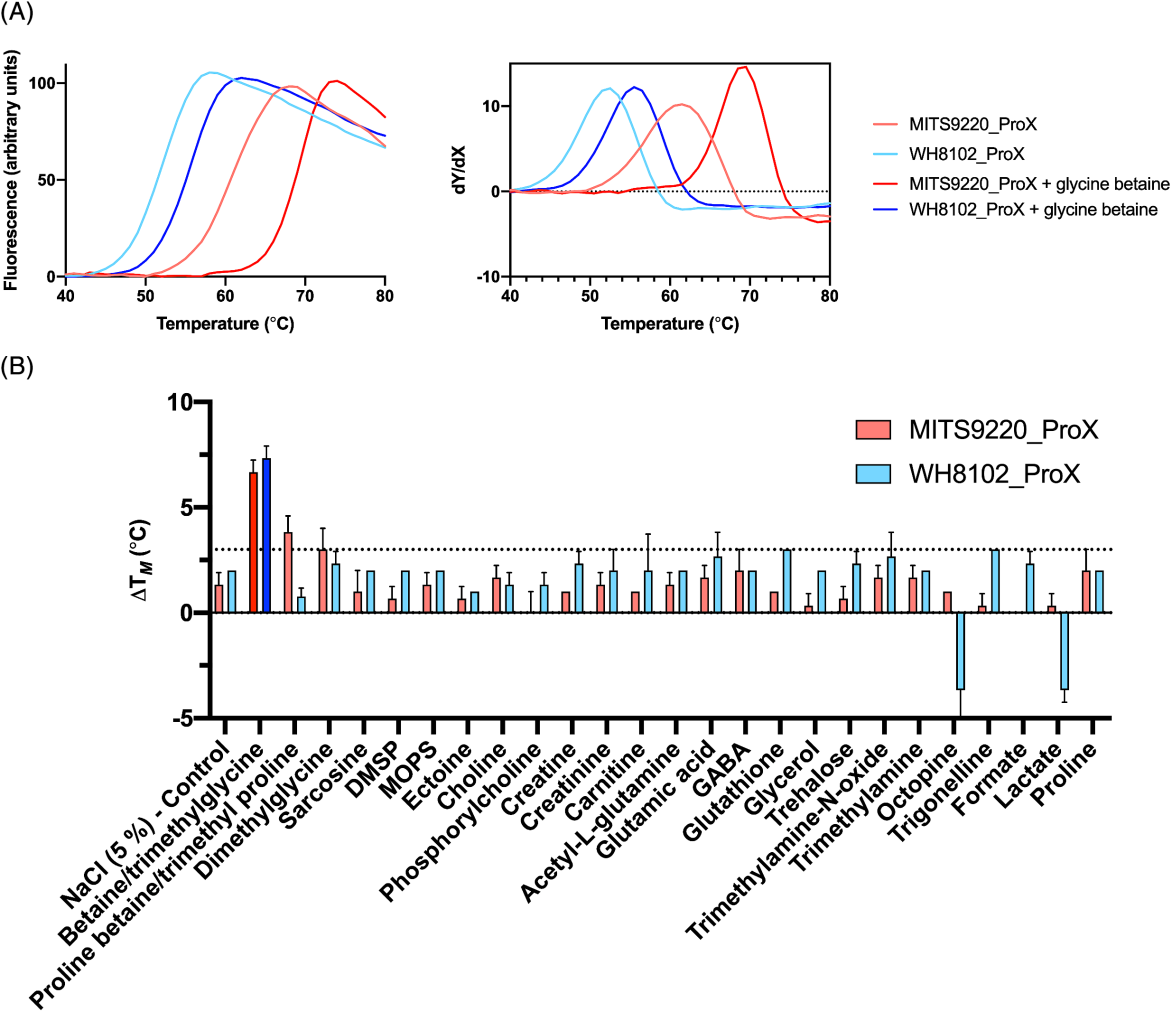
Ligand screening of MITS9220_ProX and WH8102_ProX. (A) The normalized thermal unfolding curves (left) and first derivative (right) for purified MITS9220_ProX (red) and WH8102_ProX (blue), both as native proteins and in the presence of glycine betaine, which causes a shift in the observed melting temperature (T_*M*_). (B) A range of common osmoprotectants tested against MITS9220_ProX (red) and WH8102_ProX (blue) to identify alternative ligand candidates. These data show that the only significant change in melting temperature (ΔT_*M*_) is conferred by glycine betaine (trimethyl glycine).

Both WH8102_ProX and MITS9220_ProX were subjected to thermal melt analysis by differential scanning fluorimetry (DSF) to derive their melting temperatures (T_*M*_ = 52 and 60.5°C, respectively). This was repeated in the presence of small‐molecule additives (Silver Bullets, Hampton Research) to identify putative binding partners based on changes in observed melting temperatures. One cocktail condition (Silver Bullets condition D8 containing a range of osmoprotectants: glycine betaine, l‐glutamic acid, l‐proline, taurine and trimethylamine‐N‐oxide, 0.2% w/v each) led to a marked increase in melting temperature (ΔT_*M*_ = 8°C) for MITS9220_ProX and a smaller increase (ΔT_*M*_ = 6°C) for WH8102_ProX. Deconvolution of the cocktail showed that glycine betaine (trimethyl glycine) was solely responsible for the observed change in the melting temperature (Figure 3A), consistent with their predicted ligand chemistry as glycine/proline betaine‐binding proteins. Despite not being present in any commercially available screens, proline betaine was also tested independently: WH8102_ProX showed T_M_ shifts <2°C, and MITS9220_ProX showed a minor shift of approximately 4°C, possibly indicating a weak interaction with this substrate.

To ascertain whether other osmoprotectants altered the observed T_*M*_, MITS9220_ProX and WH8102_ProX were screened against 25 known osmoprotectants (PM9 Microplate, Biolog). The screen included chemical analogs of betaine: mainly alternative quaternary amines, dimethylglycine, trimethylamine, trimethylamine N‐oxide, and so on. Both proteins displayed a shift in melting temperature in the presence of glycine betaine consistent with that expected from previous screening (∆T_*M*_ + 6–7°C). Compared to glycine betaine, a negligible change in the T_*M*_ was observed for other compatible solutes (Figure 3B). The DSF results indicate that MITS9220_ProX and WH8102_ProX strongly prefer glycine betaine and, to a small extent, proline betaine over other related osmoprotectants.

### ProX binding affinity for glycine betaine differs between Synechococcus strains

**FIGURE 4:**
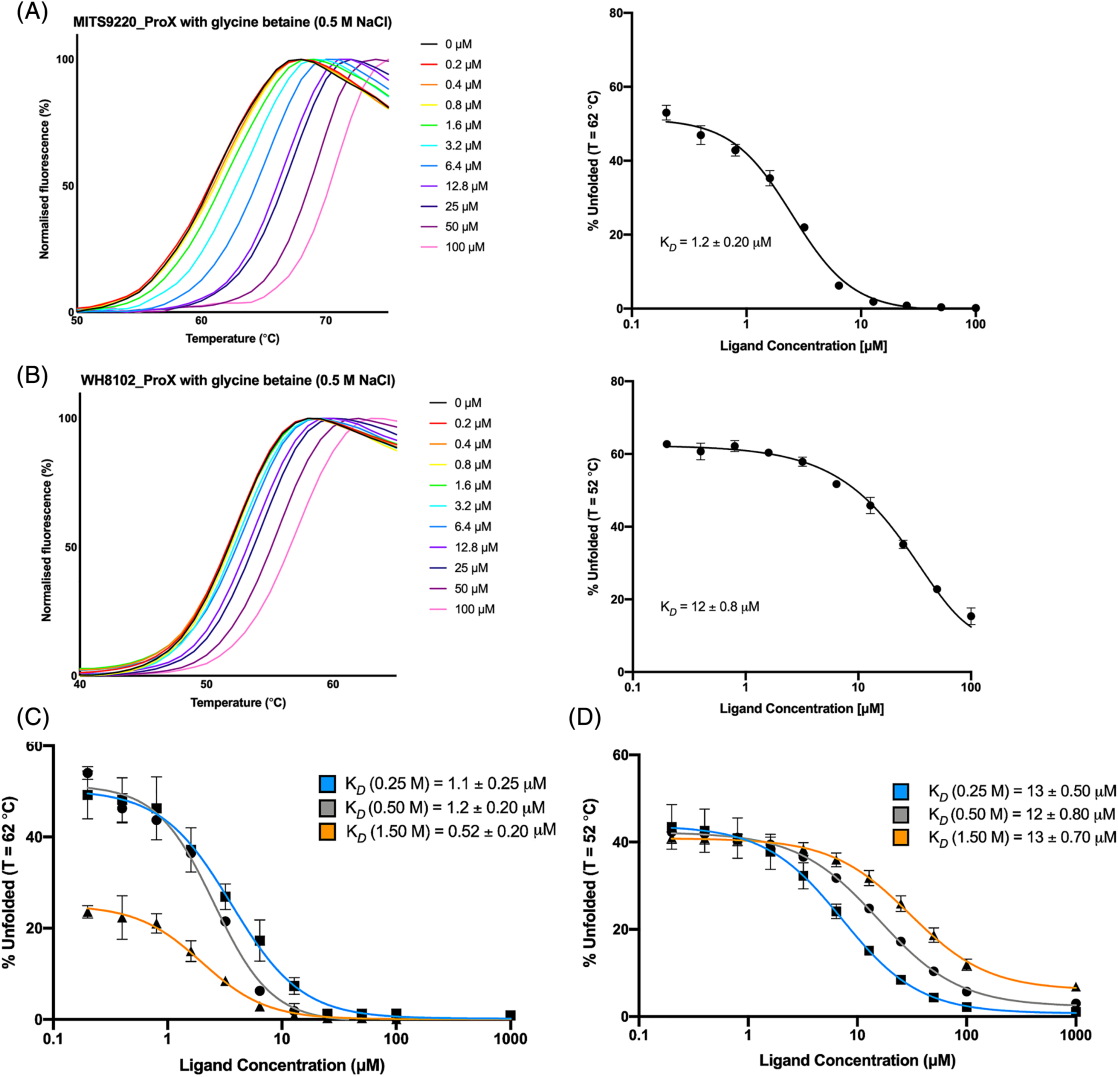
MITS9220_ProX and WH8102_ProX binding affinity to glycine betaine. Both MITS9220_ProX (A) and WH8102_ProX (B) show increasing shifts in observed melting temperatures upon the incremental addition of the glycine betaine ligand in the presence of 0.5 M NaCl. The corresponding isothermal analysis of the DSF data used to calculate the binding affinity is depicted (right). The isothermal analysis of the effect of ionic strength, in the presence of a range of salt concentrations ([NaCl] = 0.25, 0.50, and 1.5 M), on MITS9220_ProX (C) and WH8102_ProX (D) glycine betaine binding. Reported affinities were computed using the FoldAffinity server (Niebling et al., 2021).

Purified ProX samples were subjected to binding kinetics determination using a modified DSF approach (Bai et al., 2019; Niebling et al., 2021). This approach was selected due to aberrant noise in calorimetric and tryptophan quenching experiments, attributed to possible dimer‐monomer interconversion co‐occurring with ligand‐binding events. In this kinetics assay, increasing glycine betaine concentrations led to incremental shifts in the observed melting temperature of ProX (Figure 4A,B). This is related to the proportion of folded versus unfolded protein at a single temperature (isothermal analysis) using a selected temperature value (*T*_*sel*_ *)* slightly above the T_*M*_ previously determined. Using the FoldAffinity tool (Niebling et al., 2021), non‐linear regression and curve fitting of the processed thermal melt curves allowed the determination of ligand dissociation constants (K_*D*_) for each protein and ligand partner.

For MITS9220_ProX, the specific affinity for glycine betaine was found to be 1.2 ± 0.20 μM (at T_sel_ 62°C), which is comparable to most functionally related proteins described in Table 1. The measured MITS9220_ProX K_*D*_ value for glycine betaine in the low micromolar range reflects the marked thermal shift, ∆T_*M*_ = 20°C, observed in excess ligand concentrations (leading to complete formation of the protein: ligand complex). Under identical conditions, the affinity calculated between WH8102_ProX and glycine betaine was 12 ± 0.8 μM. This represents a tenfold lower affinity interaction than MITS9220_ProX, and other characterized ProX homologues (Table 1). The binding affinity of the two ProX proteins to glycine betaine in the micromolar range, in addition to the co‐occurrence of the *proU and gbmt1/gbmt2* gene clusters, is consistent with the ‘leakage recovery’ hypothesis (Scanlan et al., 2009). This suggests that the function of the ProX protein is to salvage the glycine betaine leaked through the cytoplasm into the periplasm, facilitating reuptake via the *proU* ABC machinery.

**TABLE 1:** Reported binding affinities of proteins related to *Synechococcus* ProX proteins

Organism	SBP	PDB ID	Sequence ID to MITS9220_ProX (%)^a^	Ligand	Affinity (K_*D*_) (μM)	References
*Synechococcus sp*. MITS9220	ProX	N/A	N/A	Glycine betaine (0.25 M NaCl)	1.1	This study
				Glycine betaine (0.50 M NaCl)	1.2	
				Glycine betaine (1.5 M NaCl)	0.52	
*Synechococcus sp*. WH8102	ProX	N/A	58.1	Glycine betaine (0.25 M NaCl)	13	This study
				Glycine betaine (0.50 M NaCl)	12	
				Glycine betaine (1.5 M NaCl)	13	
*Archaeoglobus fulgidus*	ProX	1SW1	17.3	Proline betaine	0.05	Schiefner, Holtmann, et al. (2004))
		1SW2				
				Glycine betaine	0.06	
		1SW4		Trimethyl ammonium	‐	
*E. coli*	ProX	1R9L	20.4	Glycine betaine	1.0	Schiefner, Breed, et al. (2004))
		1R9Q		Proline betaine	5.0	
*Sinorhizobium meliloti*	ChoX	2REG	22.3	Choline	2.7	Oswald et al. (2008)
		2RIN		Acetylcholine	145	
*L. lactis*	OpuAC	3L6G, 3L6H	28.7	Glycine betaine	4.5	Wolters et al. (2010)
				Proline betaine	41	
				Proline	‐^b^	
				Carnitine	‐^b^	
*Ruegeria pomeroyi*	TmoX	4XZ6	17.5	Trimethylamine oxide	1.6	Li et al. (2015)
				Glycine betaine	‐	
				Choline	3.8	
				Carnitine	‐	

^a^ Identity to mature sequence.

^b^ Binding detected; however, K_*D*_ is too large to be physiologically relevant (>10 mM).

Similar kinetic measurements using proline betaine showed affinity values that were several orders of magnitude lower. For MITS9220_ProX, this affinity was negligible (K_*D*_ > 1000 μM), and for WH8102_ProX, it was found to be 250 ± 80 μM (~200‐fold lower than glycine betaine) (Figure S3). This work indicates that glycine betaine is most likely the natural substrate of both MITS9220_ProX and WH8102_ProX, and by extension, also of the other CK_1944 members. The differences in the binding affinity of *Synechococcus* ProX isolated from two discrete phylogenetic groups (see Figure 1A) also functionally explain their delineation into two distinct protein clades that likely correspond to unique eco‐physiological adaptations.

### Ligand binding of MITS9220_ProX is affected by salt concentration

Both initiation of transcription of compatible solute‐binding protein genes and translocation activity of the entire uptake machinery are caused by changes in the ionic strength of the growth medium (Van Der Heide & Poolman, 2000). We investigated whether changes in the ionic strength of handling buffers might affect the protein activity. This hypothesis has previously been tested in OpuAC from *L. lactis*, which indicated that changing salt concentrations did not influence the binding kinetics (Wolters et al., 2010).

An unresolved question from the solution‐state analysis was whether salt concentrations meaningfully affect the solution properties of ProX, thereby affecting its activity. Having identified glycine betaine as the ligand of both MITS9220_ProX and WH8012_ProX using 0.5 M NaCl (Figure 4A,B), the DSF kinetics assay was repeated using different NaCl concentrations: 0.25 M (low salt) and 1.5 M (high salt). For MITS9220_ProX, these normalized melting curves show apparent differences between the melting temperatures obtained for low‐ and high‐salt conditions, with high‐salt conferring additional thermal stability (ΔT_*M*_ = 5°C). In comparison, for WH8102_ProX, increasing salt concentrations led to slight destabilization of the protein, indicated by a small reduction in observed melting temperature (ΔT_*M*_ = −2°C).

Plotting the proportion of folded versus unfolded protein at the T_*M*_ of MITS9220_ProX (Figure 4C), it was clear that the high‐salt condition conferred higher thermal stability compared with both the low‐ and moderate‐salt conditions. For WH8102_ProX, there was negligible change in the thermal stability as salt concentrations increased (Figure 4D). Derived dissociation constants showed that for MITS9220_ProX, the high‐salt condition resulted in an altered binding affinity, corresponding a to K_*D*_ = 0.52 ± 0.20 μM, two‐fold higher than that of low salt (K_*D*_ = 1.1 ± 0.25 μM) (Figure 4C). In contrast, for WH8102_ProX, no apparent change to the dissociation constant was detected (Figure 4D).

Given that for functional relatives of MITS9220_ProX, ionic strength has not been shown to influence the binding kinetics (Wolters et al., 2010), it is surprising to see the influence of salt concentration on the binding affinity of MITS9220_ProX for glycine betaine. This represents a clear functional difference between MITS9220_ProX and currently characterized relatives, including the related *Synechococcus* protein WH8102_ProX. Physiologically, the capacity to bind compatible solutes with high affinity under high ionic strengths is justifiable. However, the conditions trialled represent a significantly higher ionic strength than expected for most marine environments (Roy et al., 2001). Therefore, the true ecological significance of this finding remains unclear. It is worth noting that binding kinetic analyses were only conducted with differing concentrations of NaCl and could be expanded to cover other candidate ions from the Hofmeister series (Papageorgiou & Murata, 1995; Zhang & Cremer, 2006) to determine whether this behaviour is consistent across different chaotropes or lyotropes due to their differing ability to alter the hydration shell of proteins.

### Synechococcus ProX structure predictions depict a modified ligand‐binding pocket

**FIGURE 5:**
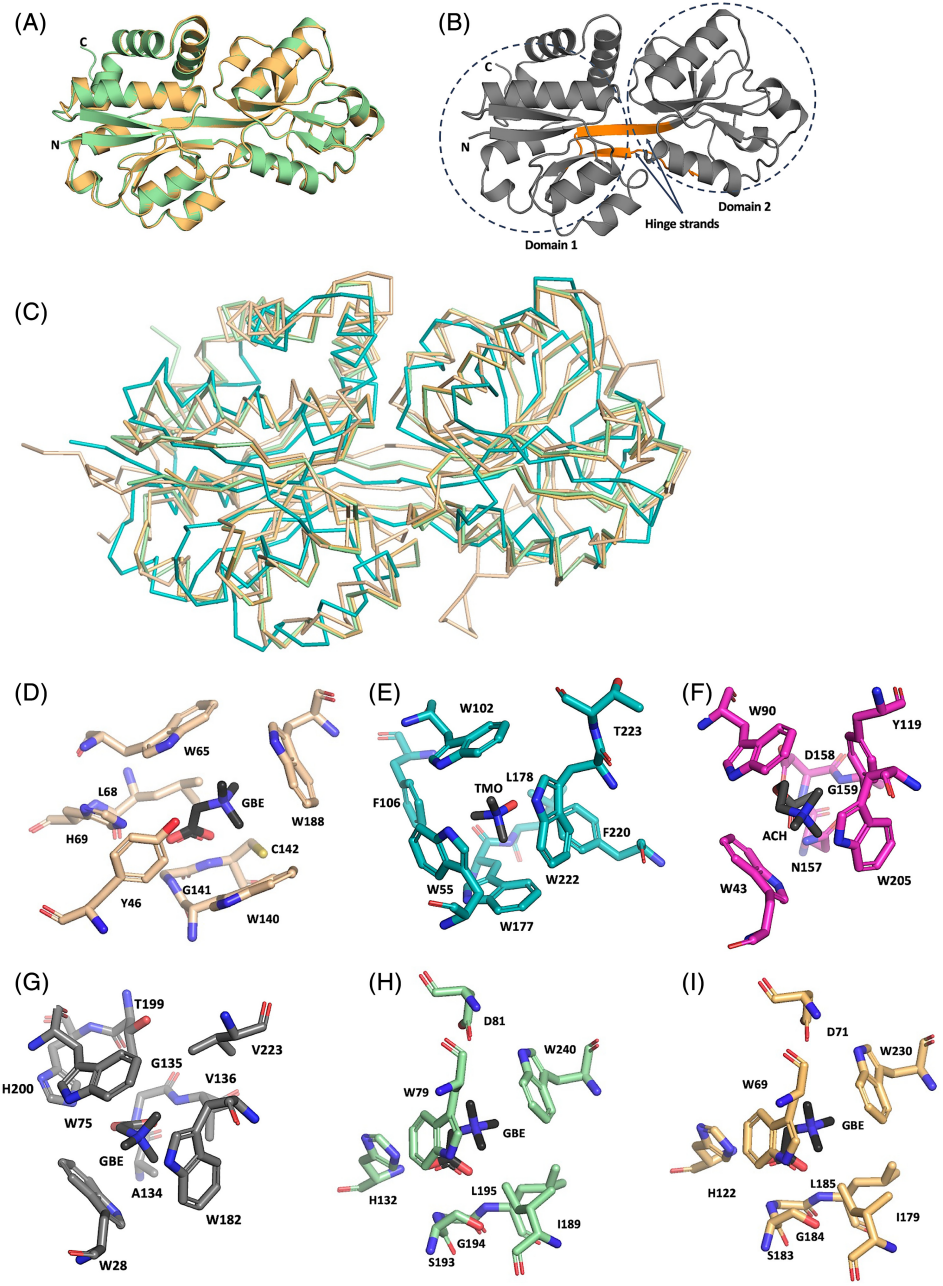
Comparison of MITS9220_ProX and WH8102_ProX predicted structures with structural relatives. (A) The predicted structures of MITS9220_ProX (green) and WH8102_ProX (orange) are shown as cartoon representations. (B) The overall structural architecture of two α/β Rossmann domains with the two elongated hinge strands (orange), which define the Cluster F‐I grouping (Berntsson et al., 2010; Scheepers et al., 2016). (C) The overlay of ribbon structures of MITS9220_ProX (green), WH8102_ProX (orange), *E. coli* ProX (teal, PDB ID 1R9L), and *L. lactis* OpuAC (wheat, PDB ID 3L6G). The root mean standard deviation of Cα backbones against the MITS9220_ProX structure is 0.279, 3.841, and 1.904 Å, respectively. Details of the ligand‐binding residues for ProX structural homologues, highlighting the tryptophan ‘cage’ used to coordinate ligands is, depicted for (D) *E. coli* ProX (wheat, PDB ID: 1R9Q) with glycine betaine (GBE) (Schiefner, Breed, et al., 2004) (E) *R. pomeroyi* TmoX (teal, PDB ID: 4XZ6) with trimethylamine‐N‐oxide (TMO) (Li et al., 2015) (F) *S. meliloti* ChoX (pink, PDB ID: 2RFN) with acetylcholine (ACH) (Oswald et al., 2008), and (G) *L. lactis* OpuAC (grey, PDB ID: 3L6G) with GBE (Wolters et al., 2010). The ligand‐binding pocket for the predicted structures of MITS9220_ProX and WH8102_ProX is presented as panels (H) and (I), respectively, with the ligand GBE overlaid from the *E. coli* ProX structure.

Structural models for WH8109_ProX and MITS9220_ProX were predicted using AlphaFold (Jumper et al., 2021) to determine their relationship to validated glycine‐betaine‐binding proteins and identify specific features that could give further insights into their function. Both predicted ProX structures display the characteristic SBP tertiary fold, consisting of two α/β Rossmann domains oriented around a flexible hinge region (Figure 5A,B). The architecture of the hinge region comprises two elongated strands, characteristic of structural class F (Berntsson et al., 2010; Scheepers et al., 2016), which encompasses the known examples of compatible solute‐binding proteins, indicating a favourable agreement between the predicted models and experimental observations. There is a high degree of structural conservation between the two *Synechococcus* ProX predicted structures (Figure 5C), with an RMSD of 0.279 Å based on the Cα backbone. This structural similarity extends to other validated compatible solute‐binding proteins, with RMSD < 1 Å to the archetypal homologue from *E. coli* and its nearest sequence relative from *L. lactis*.

Among *Synechococcus* ProX structural relatives (Table 2), the canonical ligand‐binding pattern can be described as a ‘hydrophobic cage’, where three or four aromatic residues (tryptophan or tyrosine) stabilize the quaternary amine of the ligand through cation–π interactions (Figure 5D–G). Several additional hydrophilic sidechains stabilize the carboxyl group. One of these stabilizing hydrophilic residues is typically a charged sidechain (histidine or aspartic acid), which may be necessary for mediating the association/dissociation of the ligand–protein complex. From comparing the key binding residues for the *Synechococcus* ProX structural relatives, it is apparent that they follow the canonical binding signature expected for this grouping, with most favouring the use of tryptophan to engage the ligand via cation–π interactions. More broadly, for bulkier ligands such as trimethylamine oxide, there is additional stabilization of these hydrophobic sidechains through π–π stacking from further hydrophobic sidechains embellishing the binding cleft, such as the additional phenylalanine residues observed for TmoX from *R. pomeroyi* (Figure 5E).

**TABLE 2:** Ligand coordinating residues for ProX proteins

Organism	SBP	PDB ID	Ligand	Experimental or predicted ligand‐binding residues^a^						
				R_1_	R_2_	R_3_	R_4_	R_5_	R_6_	R_7_
*Synechococcus* sp. MITS9220	ProX	N/A^b^	Glycine betaine	W79	D81	H132^c^	I189	G194	L195	W240
*Synechococcus* sp. WH8102	ProX	N/A^b^	Glycine betaine	W69	D71	H122^c^	I179	G184	L185	W230
*A. fulgidus*	ProX	1SW2	Glycine betaine	Y63	D109^c^	Y111	R149	Y190	Y214	
*E. coli*	ProX	1R9L	Glycine betaine	W65	H69^c^	W140	G141	C142	W188	
*S. meliloti*	ChoX	2REG	Choline	W43	D45^c^	W90	Y119	N156	D157	W205
*L. lactis*	OpuAC	3L6G	Glycine betaine	W330	W337	H392^c^	G437	V438	W484	
*R. pomeroyi*	TmoX	4XZ6	Trimethylamine oxide	W55	W102	F106	E122^c^	W177	F220	W222

^a^ Residues for the predicted structures from *Synechococcus* spp. are numbered according to their position in the full‐length sequence.

^b^ Structure predicted using AlphaFold (Jumper et al., 2021).

^c^ Denotes residues responsible for stabilizing the carboxyl group of the quaternary amine ligand.

In contrast, the two predicted *Synechococcus* ProX structures (Figure 5H,I) indicate that they may not have the typical binding pocket expected for this grouping. Both ProX predicted structures show only two tryptophan residues in the binding cleft. The additional residue contributing to the hydrophobic cage appears to be a leucine, which may mediate a hydrophobic interaction between the ligand, while being stabilized by a nearby isoleucine sidechain. Neither leucine nor isoleucine can engage in π–π bonding interactions, meaning the ligand may not be sufficiently stabilized by the expected cation–π interactions. While this may represent a limitation of the predicted structure, it is worth noting that other important sidechains in the binding cavity appear to fulfil their expected role, such as the observed histidine, which engages the carboxyl group of the quaternary amine.

Overall, the reduced number of hydrophobic residues, combined with an additionally charged residue (aspartic acid) predicted in the binding cavity of the predicted *Synechococcus* ProX structures, may indicate a much less hydrophobic pocket, diverging from the canonical mode, which instead requires a more extensive water‐mediated ligand‐binding network. The latter might further explain the high specificity of *Synechococcus* ProX to glycine betaine.

## CONCLUSIONS

Bioinformatics analyses indicate that some sequenced *Synechococcus* and *Prochlorococcus* strains possess uptake machinery (ProX) for acquiring compatible solutes such as betaine, likely as a strategy for alleviating osmotic stress. Many compatible solute‐binding proteins interact with chemically similar substrates (quaternary amines), with ligand binding largely determined by cation‐pi interactions between conserved aromatic residues and the residual charge from the amine group, requiring substrate preferences to be empirically determined.

Our ligand screening of MITS9220_ProX indicates a strong preference for glycine betaine, with binding kinetic data confirming a strong affinity in the micromolar range. The MITS9220_ProX affinity for glycine betaine increased twofold in high‐salt concentrations, suggesting there are subtle functional differences in solutions of different ionic strength, reflective of the required capacity to respond to high and fluctuating salt conditions across a range of environments. Therefore, it is evident that the proposed function of MITS9220_ProX is a generalist‐style compatible solute‐binding protein, consistent with its broad distribution across large regions of the marine environment.

In contrast, while WH8102_ProX displayed a strong preference for glycine betaine, the kinetic data indicated a comparatively lower affinity than MITS9220_ProX. Also, the ligand‐binding behaviour of WH8102_ProX did not show any salt dependence. This shows a much more specialized compatible solute‐binding protein for relatively higher salinity environments such as the Mediterranean sea.

Our work further adds to a growing repertoire of SBPs with additional functionality, such as distinctive dimerization of *Synechococcus* ProX in solution, leaving an outstanding question about what role, if any, salt and quaternary organization play in the mechanics of ligand‐binding for ProX.

## AUTHOR CONTRIBUTIONS

Conceptualization, Benjamin A. Ford, Bridget C. Mabbutt, Ian T. Paulsen, and Bhumika S. Shah; Methodology, Benjamin A. Ford, Pramita Ranjit, and Bhumika S. Shah; Formal Analysis, Benjamin A. Ford, Ian T. Paulsen, and Bhumika S. Shah; Writing‐Original draft, Benjamin A. Ford, and Bhumika S. Shah; Writing‐reviewing and editing, Benjamin A. Ford, Ian T. Paulsen, and Bhumika S. Shah.

## CONFLICT OF INTEREST

The authors declare no conflict of interest.

## Supporting information


**Figure S1** Geographic distribution of MITS9220 and WH8102 *proX* homologues (MetaT).
**Figure S2**. Analytical SEC traces of MITS9220_ProX and WH8102_ProX.
**Figure S3.** Binding kinetics of *Synechococcus* ProX proteins with proline betaine.
