# Supplementary material for: ProX from marine Synechococcus spp. show a sole preference for glycine‐betaine with differential affinity between ecotypes
Source: Environ Microbiol. 2022 Aug 28;24(12):6071–85. doi: 10.1111/1462-2920.16168 (PMC10087775; doi:10.1111/1462-2920.16168)
Supplement: Supplementary file 1 — Figure S1 Geographic distribution of MITS9220 and WH8102 proX homologues (MetaT). Figure S2. Analytical SEC traces of MITS9220_ProX and WH8102_ProX. Figure S3. Binding kinetics of Synechococcus ProX proteins with proline betaine. [file EMI-24-6071-s001.pdf]

Supplementary Figures

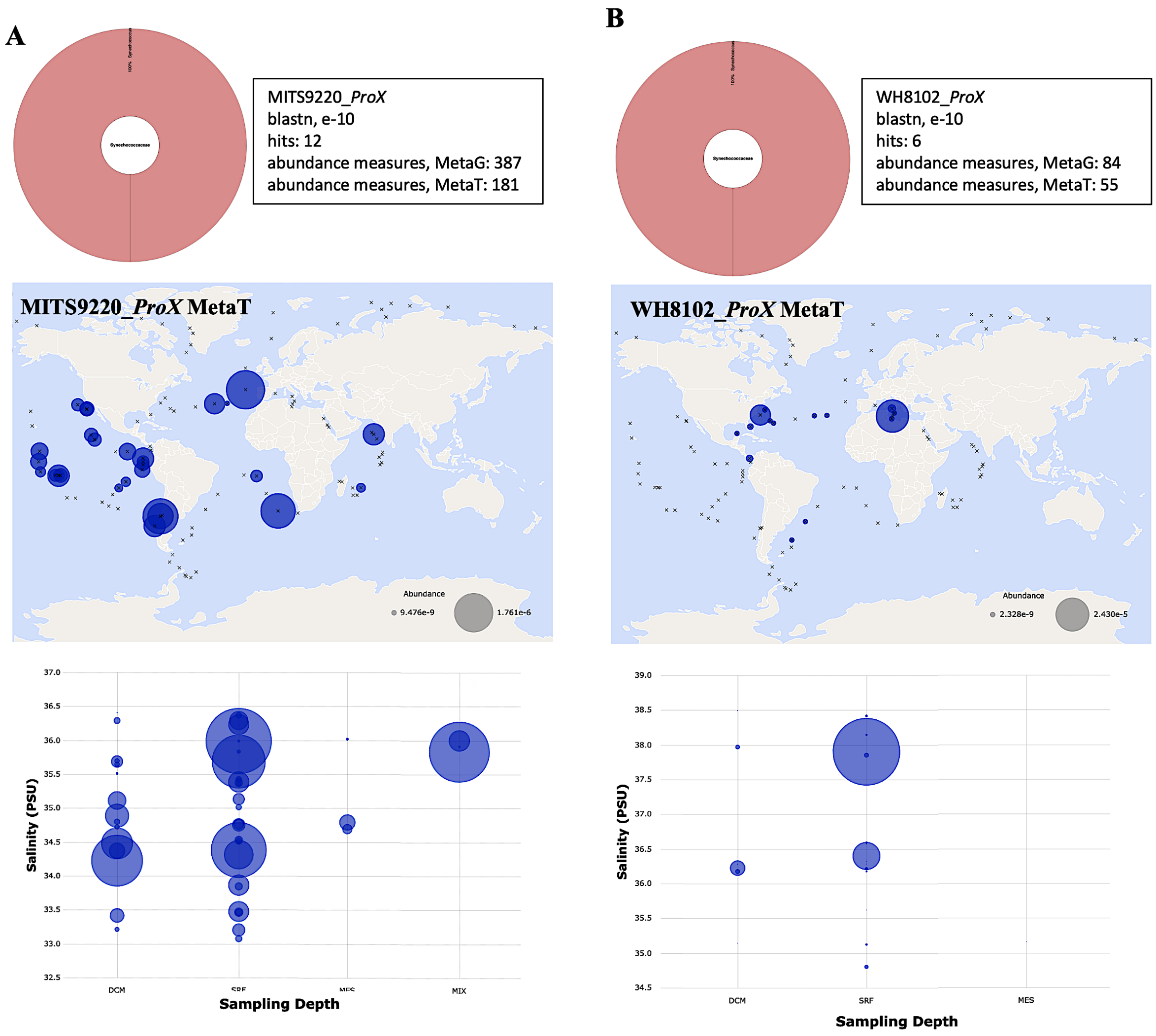

Figure S1: Geographic distribution of MITS9220 *proX* and WH8102 *proX* homologs

Top, Krona plot depicting the taxonomic distribution of **(A)** MITS9220_*ProX* and **(B)** WH8102_*ProX* homologs (>75% nucleotide sequence identity) selected to analyse the MetaG and MetaT abundance measures (Sunagawa *et al.* 2020). Middle, transcript abundance for **(A)** MITS9220_*ProX* and **(B)** WH8102_*ProX* homologs extracted from the Tara Oceans MetaT dataset. The abundance is plotted for surface waters, with circle size corresponding to the measured abundance at a particular sampling site (denoted by an ‘x’). Bottom, the corresponding bubble plot across all four sampling depths as a function of measured salinity for **(A)** MITS9220_*ProX* and **(B)** WH8102_*ProX* homolog transcripts.

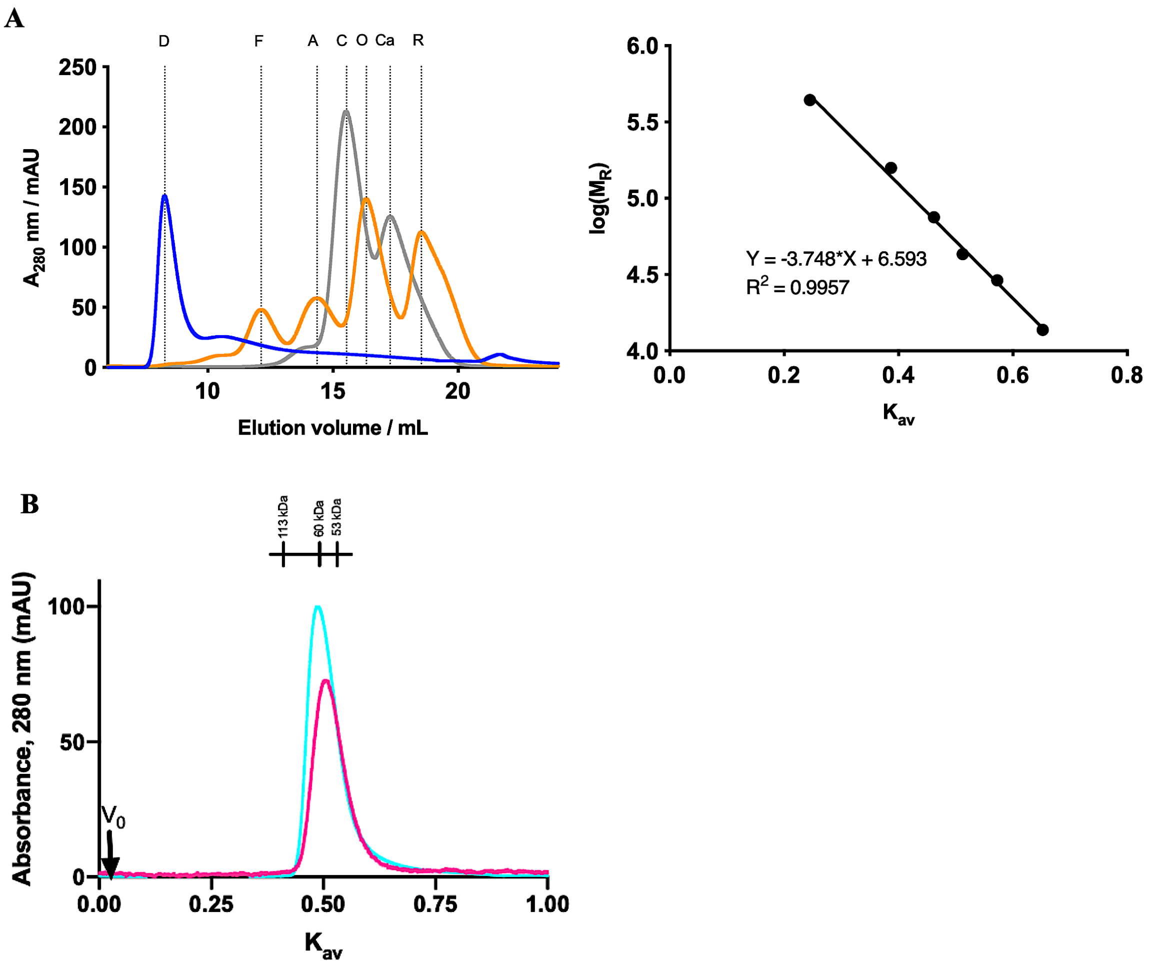

Figure S2: Analytical SEC traces of MITS9220_ProX and WH8102_ProX.

(A) Left, standards ferritin (F), aldolase (A), conalbumin (C), ovalbumin (O), carbonic anhydrase (Ca) and RNase (R) of known M_R were used to calibrate the Superdex 200 10/300 GL column (GE-Healthcare), equilibrated in buffer containing HEPES (50 mM, pH 7.4), NaCl (500 mM), and glycerol (5% v/v) for analytical SEC. The void volume (V_0) was estimated using blue dextran (D). Right, a calibration plot showing calculated K_{av} and logarithm of molecular radius used to interpolate the size of each ProX protein sample. (B) Analytical SEC of MITS9220_ProX (cyan) and WH8102_ProX (magenta) showing single solution-state consistent with an apparent molecular weight of a dimer.

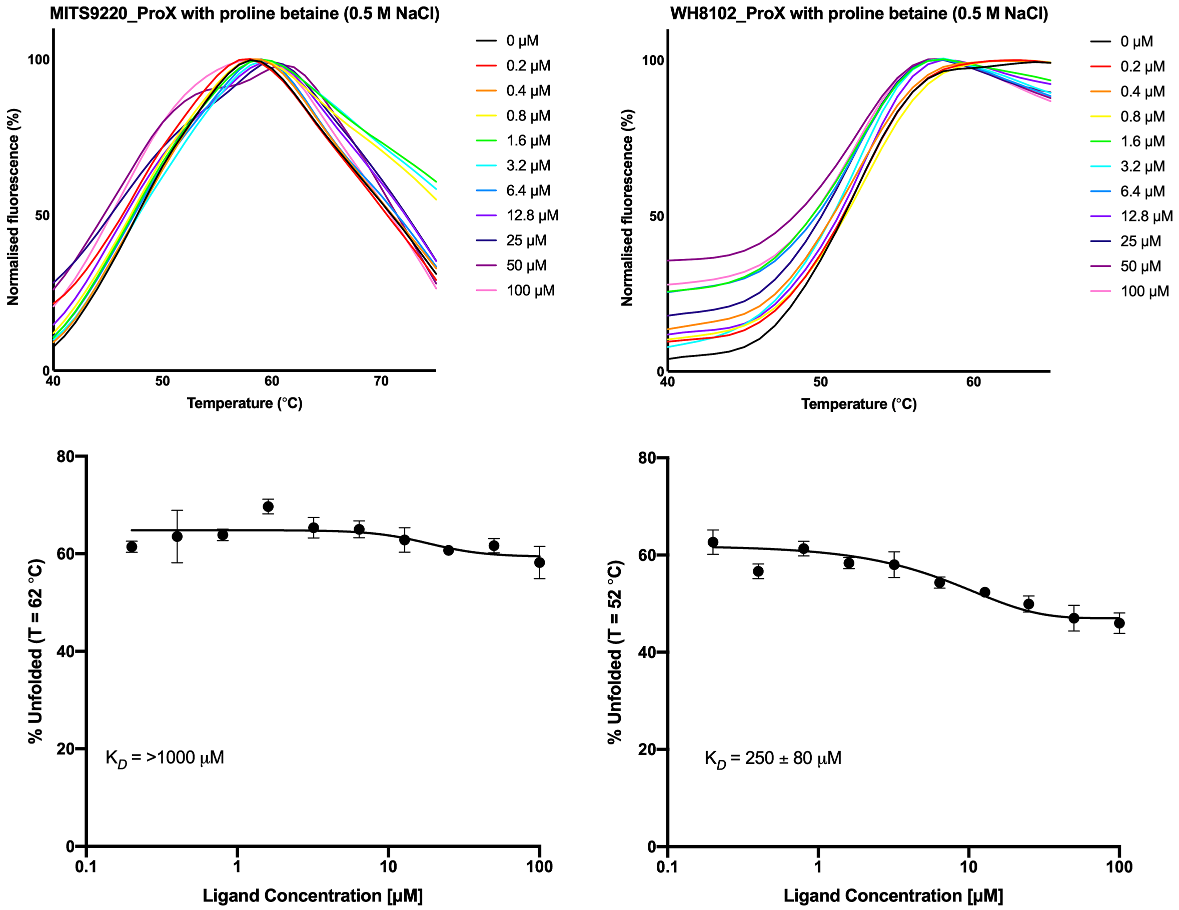

Figure S3: Isothermal analysis of *Synechococcus* ProX proteins with proline betaine. Above, the thermal shift curves from the modified DSF experiment used to calculate the binding affinity in the presence of proline betaine. Both MITS9220_ProX (left) and WH8102_ProX (right) show negligible shifts in observed melting temperatures upon the incremental addition of the proline betaine. Below, the corresponding fraction of unfolded proteins and associated curve fitting used to calculate the binding of proline betaine with MITS9220_ProX (left) and WH8102_ProX (right).
